# Tuberculosis trend among native and foreign-born people over a 17 year period (2004–2020) in a large province in Northern Italy

**DOI:** 10.1038/s41598-021-02540-4

**Published:** 2021-12-03

**Authors:** Valentina Marchese, Luca Rossi, Beatrice Formenti, Michele Magoni, Anna Caruana, Claudio Sileo, Laura Lanfredini, Francesco Castelli, Alberto Matteelli

**Affiliations:** 1 Department of Infectious and Tropical Diseases, ASST Spedali Civili of Brescia, University of Brescia, Piazzale Spedali Civili 1, 25123 Brescia, Italy; 2 WHO Collaborating Center for TB/HIV Co-infection and the TB Elimination Strategy, Department of Clinical and Experimental Sciences, University of Brescia, Brescia, Italy; 3 UNESCO Chair “Training and Empowering Human Resources for Health Development in Resource-Limited Countries”, University of Brescia, Brescia, Italy; 4 Brescia Health Protection Agency (ATS), Brescia, Italy

**Keywords:** Epidemiology, Tuberculosis

## Abstract

Tuberculosis (TB) incidence should decline by 20% in the Europe in 2015–2020, in line with End-TB milestones. We retrospectively evaluated TB notifications in the province of Brescia from 2004 to 2020. Cases were classified per patient origin and entitlement to Health Assistance for foreign born people: Italians (ITA), Foreigners permanently entitled (PEF) or Temporarily Entitled (TEF) to Health Regional Assistance. Poisson regression analysis was performed to assess associations between incidence and age, sex, continent of origin and year of notification. Overall 2279 TB cases were notified: 1290 (56.6%) in PEF, 700 (30.7%) in ITA and 289 (12.7%) in TEF. Notifications declined from 15.2/100,000 in 2004 to 6.9/100,000 in 2020 (54.6% reduction, temporary increase in 2013–2018 for TEF). Age (Incidence Risk Ratio, IRR, 1.02, 1.019–1.024 95%CI), sex (IRR 1.22, 1.12–1.34 95%CI), and continent of origin were positively associated with notifications (IRR 34.8, 30.8–39.2 95%CI for Asiatic, and IRR 20.6, 18.1–23.4 95%CI for African origin), *p* < 0.001. Notification decline was sharper in 2020, especially among TEF. End-TB milestone for 2020 was reached, but foreigners continue to represent a high risk group for the disease. Discontinuation of services due to the COVID-19 pandemic was associated with a sharp decrease in TB notification in 2020.

## Introduction

Tuberculosis (TB) is still a major public health concern in the WHO European Region, including the countries of the European Union/European Economic Area (EU/EEA). In 2019 246,000 incident cases were notified to the European Centre for Diseases Control and Prevention (ECDC), corresponding to a rate of 26 cases (23–30) per 100,000 population^1^.

The target of the Sustainable Development Goals agenda for TB is to end the epidemic by 2030. In EU/EEA this means achieving 2.4/100,000 TB cases in 2030 (80% reduction compared to 2015) and reducing TB incidence by 20% against the 2015 baseline in 2020^2^. In the WHO European region the notification rate decreased by 19% in the period 2015–2019, getting very close to the 2020 End TB Strategy milestone^1^.

In 2020 several high TB burden countries observed substantial reductions in TB notification after the beginning of the COVID-19 pandemic^2^. According to modelling studies, a reduction of 25% of diagnosis of TB would determine a 26% increase in TB deaths, with a down step of TB mortality to that estimated in 2012^3^.

In Italy, the TB incidence was 5.5/100,000 in 2019, confirming the decline observed over the previous years^1^. The majority of TB cases were detected in foreign-born people (56.2%), as seen in other low incidence TB countries^4^.

In the province of Brescia, in the North of Italy, censed foreign born people account for 12.1% of the population (1,200,000 province population)^5^, while undocumented migrants are estimated to be 8.8% of the foreigners. The presence of foreigners in Brescia is higher than at national level, where migrants represent 8.4% of the resident population^6^. Since March 2020 Brescia became one of the provinces most affected by the Coronavirus disease (COVID-19) epidemic in Europe, with a contagion rate of 8.2% (up to 10th May 2021)^7^. It was one of the very first Italian areas to implement contagion control measures in the early epidemic, including the interruption of the majority of outpatient consultations, screening and diagnostic services, as well as dedicated migrant health clinics, due to the reallocation of health resources.

In this study we describe the trend of TB notification in the Province of Brescia over a 17-year period.

## Methods

We conducted a retrospective analysis of TB notifications in the province of Brescia, Northern Italy from 1^st^ January 2004 to 31st December 2020.

All TB cases notified to the Health Protection Agency (ATS) in Brescia through the Regional Notification System were included in the analysis. Cases notified more than one time within 12 months were included using first notification, unless they had an outcome report of treatment completion or TB cure within the same 12-month period, as suggested per National Notification System^8^.

Cases were classified per patient origin and entitlement to Health Assistance for foreign born people: Italians (ITA), Foreigners permanently entitled to Health Regional Assistance (PEF) and Temporarily Entitled Foreigners (TEF). Specifically, foreigners not registered in the Regional Health System during the previous year were classified as TEF. TB cases among Italians not residing in the study area were excluded.

TB notification rate per year was calculated using the Regional Statistical Residence Data for ITA and PEF through permanent census (ISTAT), and from estimates of irregular foreigners’ presence for TEF^9–11^. Distribution per sex, country of origin and age were evaluated only for ITA and PEF (residing population), reliable estimates not being available for TEF. Rates of total population stratified by sex and 5-years age groups were used to calculate expected number over observed ones, and adjusted rates were produced using STATA software (Stata 16.1, Copyright 1985–2019 StataCorp LLC Statistics/Data analysis StataCorp 4905 Lakeway Drive Special Edition College Station, Texas 77845 USA 800-STATA-PC, https://www.stata.com). The adjusted rates could not be calculated for the TEF.

Multivariate Poisson regression analysis was performed to assess association between TB incidence and age, sex, continent of origin and year of notification. *P*-values lower than 0.05 were considered statistically significant. Results were expressed as incidence rate ratio (IRR) and their 95% confidence intervals (95% CIs).

The study protocol received ethical approval from the competent Ethics Committee (Comitato Etico Provinciale di Brescia, protocol study number 4337). It was conducted in agreement with the Declaration of Helsinki, the ICH Harmonized Tripartite Guidelines for Good Clinical Practice principles, and the Italian legislation requirements. Informed consent was obtained from all subjects or parents and/or legal guardians, when appropriate. Waiver on informed consent was obtained from the competent Ethics Committee (Comitato Etico Provinciale di Brescia) for patients not anymore in clinical follow up or lost to follow up.

## Results

During the study period, 2279 TB cases were notified: 700 (30.7%) were reported among Italians, 1290 (56.6%) in PEF, and 289 (12.7%) in TEF. Overall, foreign-born cases accounted for 69.3% of total cases (Table 1).

**Table 1:** Sex and age distribution of TB cases and relative crude and adjusted rates per ITA, PEF and TEF.

N (%)	ITA 700 (30.7)	PEF 1290 (56.6)	TEF 289 (12.7)	Total 2279
Male N (%)	389 (55.6)	762 (59.1)	219 (75.8)	1370 (60.1)
Mean age, years (SD)	57.6 (22.4)	33.9 (13.9)	32.3 (12.1)	41.0 (12.1)
Age distribution (years)	N (%)			
00–04	15 (2.1)	37 (2.9)	3 (1.0)	55 (2.4)
05–09	8 (1.1)	11 (0.9)	2 (0.7)	21 (0.9)
10–14	6 (0.9)	45 (3.5)	3 (1.0)	54 (2.4)
15–19	19 (2.7)	78 (6.0)	16 (5.5)	113 (5.0)
20–24	17 (2.4)	149 (11.6)	52 (18.0)	218 (9.6)
25–29	30 (4.3)	199 (15.4)	69 (23.9)	298 (13.1)
30–34	28 (4.0)	184 (14.3)	58 (20.1)	270 (11.8)
35–39	31 (4.4)	208 (16.1)	31 (10.7)	270 (11.8)
40–44	48 (6.9)	143 (11.1)	11 (3.8)	202 (8.9)
45–49	52 (7.4)	98 (7.6)	16 (5.5)	166 (7.3)
50–54	57 (8.1)	56 (4.3)	10 (3.5)	123 (5.4)
55–59	30 (4.3)	33 (2.6)	8 (2.8)	71 (3.1)
60–64	38 (5.4)	15 (1.2)	6 (2.1)	59 (2.6)
65–69	57 (8.1)	15 (1.2)	1 (0.3)	73 (3.2)
70–74	61 (8.7)	6 (0.5)	2 (0.7)	69 (3.0)
75–79	80 (11.4)	6 (0.5)	0 (0.0)	86 (3.8)
80–84	72 (10.3)	5 (0.4)	1 (0.3)	78 (3.4)
≥ 85	51 (7.3)	2 (0.2)	0 (0.0)	53 (2.3)
Crude rate (CI 95%)/100.000	4.1 (3.8–4.4)	50.8 (48.8–54.5)	117.8 (104.8–132.4)	11.5 (11.1–12.0)
Adjusted rate (CI 95%)/100.000	4.2 (3.9–4.5)	45.6 (43.2–48.2)		

Crude rate: crude TB notification rate (N/100,000); Adjusted rate: sex and age adjusted TB notification rate (N/100,000); CI95%: Confidence Interval 95%. Adjusted rate for TEF not measurable due to uncertainty of reference population.

Twenty-one patients were notified two times and 2 patients 3 times (more than 12 months apart) during the study period. These patients accounted for 2 and 3 cases, respectively.

**Figure 1:**
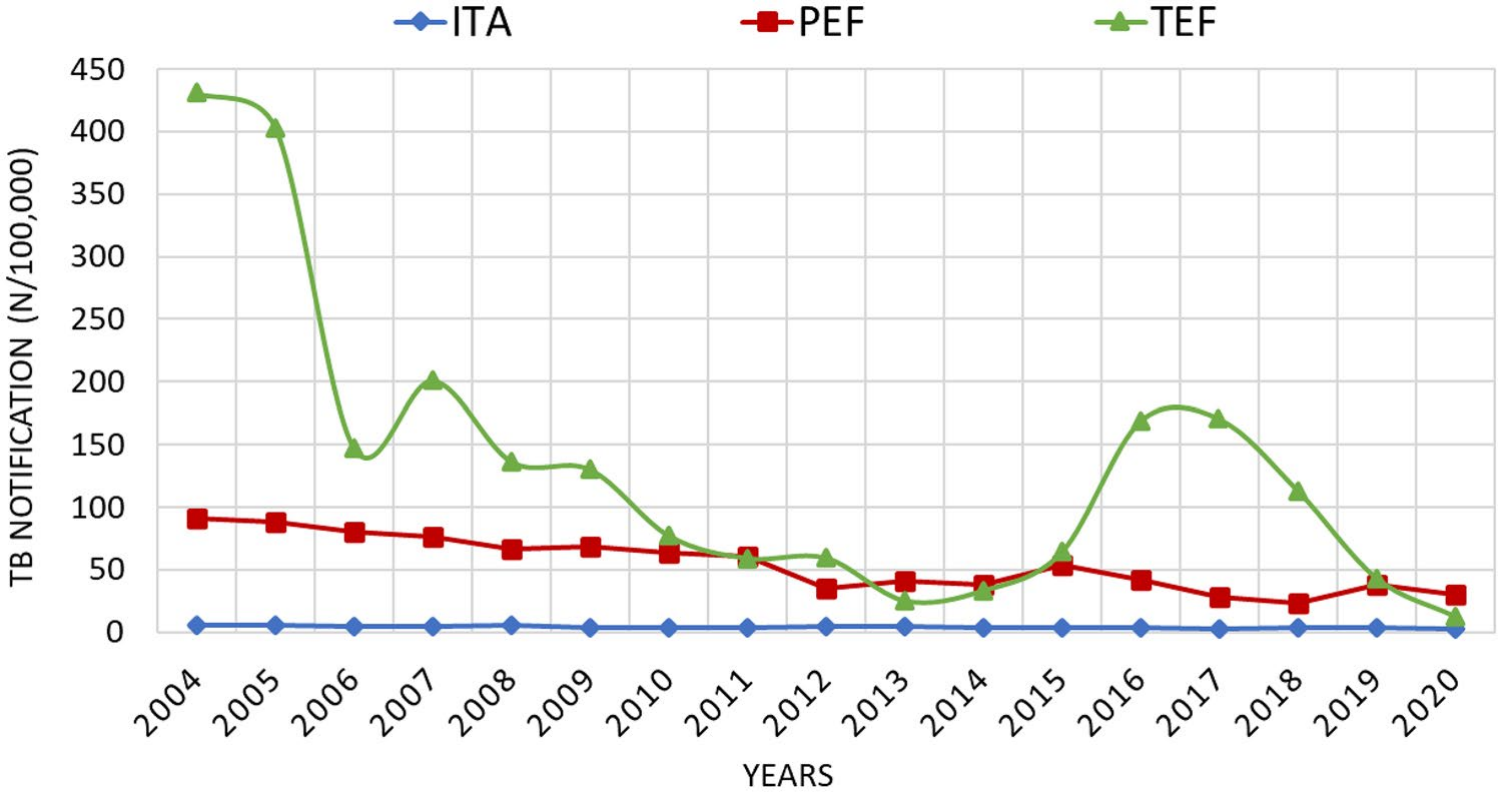
Trend of TB notification among ITA, PEF, and TEF.

Overall, TB notification rate declined from 15.2/100,000 in 2004 to 6.9/100,000 in 2020 (54.6% reduction), with differences in the three groups, being proportionally higher in foreigners compared to ITA (Fig. 1). In natives, notifications decreased by 50.8% (from 5.9/100,000 to 2.9/100,000), versus a 76.1% decline among foreign-born (from 121.0/100,000 to 28.9/100,000) (Fig. 1). During the study period TB incidence in TEF was 2.3 times higher than among PEF (117.8/100,000 vs 50.8/100,000), and 28.6 higher compared with natives (117.8/100,000/person-year vs 4.1/100,000). Sex and age adjusted rates were 45.6/100,000 (IC95% 43.2–48.2) for PEF, and 4.2 (IC95% 3.9–4.5) for ITA, respectively.

The strongest reduction in TB notification rate was registered in TEF, with a decreasing trend from 2004 to 2013, followed by an increase in 2014–2017, and a new drastic decrease from 2018 to 2020 (Table 2).

**Table 2:** TB notification trend among ITA, PEF and TEF; N: number of notified cases; Rate: n/100,000. Percentage variations in italics.

Year	ITA				PEF				TEF				Total		
	N	(%)	Rate	*var %*	N	(%)	Rate	*var %*	N	(%)	Rate	*var %*	N	Rate	*var %*
2004	58	(35.8)	5.9		71	(43.8)	90.7		33	(20.4)	430.8		162	15.2	
2005	60	(33.7)	6.1	*3.4*	79	(44.4)	88.0	* − 3.0*	39	(21.9)	403.2	* − 6.4*	178	16.5	*8.6*
2006	46	(29.7)	4.7	* − 23.0*	88	(56.8)	80.1	* − 9.0*	21	(13.5)	147.1	* − 63.5*	155	14.0	* − 15.2*
2007	45	(27.6)	4.5	* − 4.3*	91	(55.8)	76.8	* − 4.1*	27	(16.6)	200.8	*36.5*	163	14.5	*3.6*
2008	53	(31.2)	5.3	*17.8*	94	(55.3)	66.4	* − 13.5*	23	(13.5)	136.4	* − 32.1*	170	14.7	*1.4*
2009	33	(20.2)	3.3	* − 37.7*	106	(65.0)	68.5	*3.2*	24	(14.7)	130.1	* − 4.6*	163	14.0	* − 4.8*
2010	34	(23.0)	3.4	*3.0*	103	(69.6)	63.7	* − 7.0*	11	(7.4)	77.2	* − 40.7*	148	12.6	* − 10.0*
2011	35	(24.1)	3.5	*2.9*	101	(69.7)	60.8	* − 4.6*	9	(6.2)	59.1	* − 23.4*	145	12.3	* − 2.4*
2012	48	(41.4)	4.8	*37.1*	60	(51.7)	35.4	* − 41.8*	8	(6.9)	59.4	*0.5*	116	9.8	* − 20.3*
2013	50	(40.7)	5.0	*4.2*	70	(56.9)	41.3	*16.7*	3	(2.4)	25.2	* − 57.6*	123	10.4	*6.1*
2014	35	(33.7)	3.5	* − 30.0*	63	(60.6)	37.6	* − 9.0*	6	(5.8)	33.3	*32.1*	104	8.8	* − 15.4*
2015	36	(26.1)	3.6	*2.9*	92	(66.7)	54.1	*43.9*	10	(7.2)	64.4	*93.4*	138	11.6	*31.8*
2016	40	(29.9)	4.0	*11.1*	71	(53.0)	41.8	* − 22.7*	23	(17.2)	168.6	*161.8*	134	11.3	* − 2.6*
2017	30	(29.4)	3.0	* − 25.0*	47	(46.1)	27.8	* − 33.5*	25	(24.5)	170.2	*0.9*	102	8.6	* − 23.9*
2018	35	(38.0)	3.5	*16.7*	39	(42.4)	23.5	* − 15.5*	18	(19.6)	112.9	* − 33.7*	92	7.7	* − 10.5*
2019	33	(31.7)	3.3	* − 5.7*	64	(61.5)	37.9	*61.3*	7	(6.7)	43.1	* − 61.8*	104	8.7	*13.0*
2020	29	(35.4)	2.9	* − 12.1*	51	(62.2)	30.5	* − 19.5*	2	(2.4)	12.5	* − 71.0*	82	6.9	* − 20.7*
2004–2020	700		4.1	* − 50.8*	1290		50.8	* − 66.4*	289		117.8	* − 97.1*	2279		* − 54.6*
2015–2020	203		3.4	* − 19.4*	364		36.0	* − 43.6*	458		92.3	* − 80.6*	652		* − 40.5*

For the period 2015–2020, which refers to the evaluation of End TB Strategy milestone, the TB notification rate in Brescia decreased by 40.5% (11.6/100,000–6.9/100,000), with higher decrease in TEF (80.6%, 64.4/100,000–12.5/100,000), intermediate in PEF (43.6%, 54.1/100,000–30.5/100,000), and lower among natives (19.4%, 3.6/100,000–2.9/100,000).

Among foreigners, the most represented continent of origin was Asia (42.6%), followed by Africa (34.4%), Europe (13.5%), South America (2.7%), and North-Central America (0.2%). For 6.5% of notifications in foreign population the nationality could not be determined.

The TB notification rate varied by continent of origin. The highest values were reported among the people from Asia (99/100,000) and Africa (69/100,000), while the notifications among subjects from South America, Europe (almost exclusively from Romania) and North-Central America were 77/100,000, 22/100,000 and 13/100,000, respectively.

**Figure 2:**
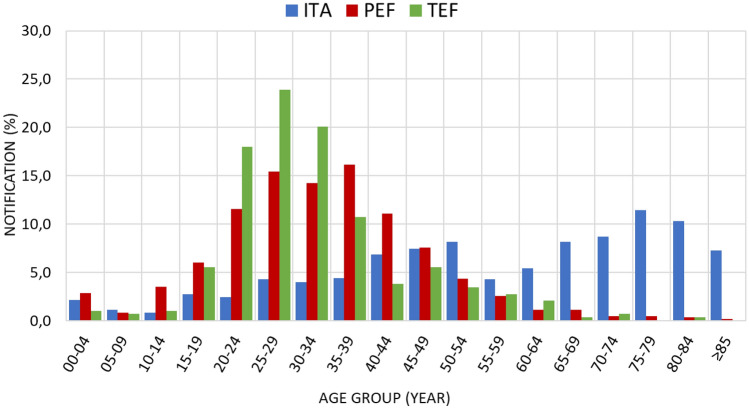
Age distribution of TB cases among ITA, PEF and TEF.

TB notifications were higher among males (60.1%), particularly among TEF (75.8%). Age distribution varied widely among groups (Fig. 2). Among Italian cases, 45.9% were over 65 years of age, with 80 (11.4%) falling in the range 75–79. In foreigners, most cases were notified among young adults. Children below 5 years accounted for 2.1%, 2.9% and 1.0% of cases among Italians, PEF and TEF, respectively (Fig. 2).

We observed a substantial reduction in cases notified in 2020 compared to 2019, at the time of the COVID-19 pandemic, in all the three populations. The greatest decline occurred in TEF (71.0%, from 43.1/100,000 to 12.5/100,000), while among PEF and ITA, the reductions were 19.5% (37.9/100,000–30.5/100,000) and 12.1% (3.3/100,000–2.9/100,000), respectively.

In the multivariable model a positive association was found between TB notifications and age (IRR 1.02), gender (IRR 1.22 for male to female) and continent of origin (IRR 34.8 and 20.6 for Asiatic and African origin, respectively), while year of notification was inversely related (IRR 0.94) (Table 3).

**Table 3:** Multivariate Poisson regression analysis of variables associated with TB diagnosis; Incidence Rate Ratio (IRR), 95% confidence intervals (95% CI) and *p*-value for age, sex, continent of origin and year of notification. Significant values are in italics.

Variable	IRR	95% CI	*p*-value
Age	1.02	1.019–1.024	< *0.001*
Sex (male to female)	1.22	1.12–1.34	< *0.001*
Year of notification	0.94	0.93–0.95	< *0.001*
**Continent of origin**			
Italians (Ref)	1		
Europe	6.7	5.6–7.9	< *0.001*
Asia	34.8	30.8–39.2	< *0.001*
Africa	20.6	18.1–23.4	< *0.001*
North-Central America	4.6	1.5–14.2	*0.008*
South America	12.9	8.4–20.0	< *0.001*
Unknown nationality	14.3	8.2–24.8	< *0.001*

## Discussion

The End TB Strategy milestone for 2020 was already reached in 2019 in the Province of Brescia, with a remarkable decline of 40.5% (11.6/100,000–6.9/100,000), in the period 2015–2020.

We observed a statistically significant association between the TB notification rate and sex, age, year of notification, and the continent of origin. Male gender predominated, as generally observed worldwide for this disease^12,13^. TB notification rate increased with increasing age: this is the result of two divergent phenomena. Most cases occurred in advanced age in Italians, while in immigrant cases most frequently occurred in younger ages, conditioned by migration demographic characteristics. Aging of the immune system is considered a driver factor for active TB among elderly, together with the lifetime cumulative increased risk of acquiring TB infection, and the presence of comorbidities which may facilitate reactivation^14^. Given the different demographic characteristics of the Italian population and migrants (mean age 45.7 years in 2020 for Italian population, 35 among migrants), TB among elderly is mostly seen in the ITA group^15^. Finally, being an immigrant (born outside Italy) was a statistically significant risk factor for TB, as observed in most settings in Europe^16^.

In our study, foreigners represented the large majority of notified TB cases (69.3% of total notifications) and had the highest notification rate. We observed crucial differences in TB risk between TEF and PEF with the former population being systematically more affected. Temporary entitled foreigners are a heterogeneous group, which includes undocumented people, asylum seekers, migrants temporarily resettled, or newly resettled during the year.

Health assistance in Italy is granted both for asylum seekers, which are temporarily entitled to the National Health System while their visa request has been processed, and undocumented migrants, which in case of health emergency have temporary entitlement to be assisted^17^.

The lower TB notification rate in PEF may be explained by the decreasing risk of TB in foreign-born populations after a long staying period in a low burden country^18,19^, as well as by the gradual improvement in health care strategies addressing migrants from high TB burden countries. Migration in the province has been constantly growing during the study period, with an almost doubled number of foreign residents from 2004 to 2020 (data extrapolated)^20^. Country of origin of foreign residents has almost remained stable across the period (Morocco, Albania and Romania), with Romania becoming the main represented country in the last decade, consistently with national data^20^. The legal process for becoming resident (and consequently permanently entitled to Health Assistance) requires time, a job position and permanent housing, by definition. All these factors are known to affect the risk of reactivation in migrants^18^.

In Italy national and regional policies encourage screening and treatment for active TB and TB infection among migrants^8,21,22^, which over time may have contributed to the steeper TB notification decline in immigrants compared to Italians. Proactive symptom-based screening for active TB is suggested both for PEF and TEF at the first medical contact. Similarly, a screening is recommended for TB infection in presence of known risk factors^8,21,22^. Given the Italian Regional Health System, those recommendations have been progressively implemented at regional level, although a national TB programme is currently not present.

For Italians and PEF the TB notification curve presents a decreasing trend throughout the study period, although isolated annual increases were registered. For ITA, the small number of TB cases is probably responsible for the high annual percentage changes. Transient increases of TB notification in PEF could be explained by changes in the distribution of country of origin, or of the employment rate at local level, as documented in 2015 (relative increased rate of Romanian origin and a growth of foreigner unemployment rate)^23^. TEF presents fluctuations within this decreasing trend. In particular, there was an evident recrudescence of cases in the period 2016–2017.

National and regional immigration reports describe a great migrant flow in 2016, with 181,000 new arrivals by sea, a number that is three times higher compared with previous years^24^. Furthermore, 2016 and 2017 saw the greatest number of asylum requests in the last decades (123,000 and 130,119) and the highest proportion of rejections was reported in 2016 (56% in Italy, reaching the proportion of 70% in Brescia)^25^. Moreover, delays were reported after the introduction of a new procedure to assign temporary fiscal codes to migrants^26^. As a consequence, asylum seekers were granted Health Assistance through the same temporary entitlement provided to undocumented migrants, and this may explain the higher notification rate reported in the same years in TEF.

Asylum seekers are known to be at higher risk for active TB if compared to other migrant populations^27,28^, with high TB incidence reported in Italian settings^29,30^. A retrospective analysis on screening for TB infection and disease conducted in Brescia among asylum seekers resettled in the period 2015–2016 found a TB prevalence at arrival of 545/100,000, and a TB incidence of 200/100,000 person/years^31^. Similar results were found in a second study performed in 2017–2018 (Marchese V, article under review).

Over the last year and a half, the COVID-19 epidemic demonstrated a significant impact on TB access to care.

In 2020 we observed a remarkable reduction of TB notifications in all the three population groups. Indeed, only 2 cases were reported among TEF, with a notification rate of 12.5/100,000 that was less than a half of the notification rate reported in PEF (30.5/100,000), and with the greatest 1-year reduction during the entire period 2004–2020. For PEF and ITA the decline was also massive in 2020 compared to 2019 (19.5%, from 37.9/100,000 to 30.5/100,000 for PEF and 12.1%, from 3.3/100,000 to 2.9/100,000 for ITA). The COVID-19 pandemic has certainly reduced people's mobility, affecting the arrivals of migrants, but not those arriving through the Central Mediterranean route, for which in 2020 an increase of landings has been reported^24^. The number of asylum requests that were filed in Italy in 2020 was 39% lower than the previous year^32^. The consequent potential higher proportion of undocumented migrants (among those arrived via Central Mediterranean route) could has been excluded from screening programs. The interruption of outpatient services, especially those dedicated to migrants, has possibly affected even more the low notification rate seen in 2020. A retrospective study performed in 2020 in Brescia found that in the early epidemic (March and April 2020) a statistically significant reduction of number of diagnoses and an increased number of deaths and losses to follow up were registered compared to the same period of 2019^33^. Diagnostic delay due to symptoms overlapping between pulmonary TB and COVID-19 or delay in seeking care due to fear of being infected in Health Care services may have negatively affected the diagnosis of active TB in 2020. Although protective measures against COVID-19 might reduce TB transmission^34^, this is not expected to have a significant effect in Italy, where the majority of cases are due to reactivation rather than recent TB infection^35,36^. We speculate that the decrease in TB notifications in 2020 does not represent a true decrease in TB incidence. Forthcoming data from 2021 will probably clarify the issue.

## Conclusion

We have found an overall decline in TB notifications during the study period, in line with the End TB Strategy milestones for 2020, but foreigners still represent the group with the highest number of cases. Migrant flows in 2016–2017 may have determined the increased notification seen in TEF in that period. Discontinuation of services due to the COVID-19 pandemic could have contributed to the reduction of notifications in 2020, especially among undocumented immigrants. The enhancement of health care strategies directed to immigrants from high TB burden countries should be a priority in the efforts towards global TB elimination.

## Data Availability

The data that support the findings of this study are available on request from the corresponding author.

## Abbreviations

TB: Tuberculosis
ATS: Health Protection Agency
ITA: Italians
PEF: Foreigners permanently entitled
TEF: Temporarily entitled
95%CI: 95% Confidence interval
IRR: Incidence rate ratio
